# Proof-of-Concept Method to Study Uncharacterized Methyltransferases Using PRDM15

**DOI:** 10.3390/ijms24021327

**Published:** 2023-01-10

**Authors:** Li-Na Zhao, Ernesto Guccione, Philipp Kaldis

**Affiliations:** 1 Department of Clinical Sciences, Lund University, P.O. Box 50332, SE-202 13 Malmö, Sweden; 2 Department of Oncological Sciences and Pharmacological Sciences, Center for Therapeutics Discovery, Tisch Cancer Institute, Bioinformatics for Next Generation Sequencing (BiNGS) Shared Resource Facility, Icahn School of Medicine at Mount Sinai, New York, NY 10029, USA; 3 Lund University Diabetes Center (LUDC), Lund University, Jan Waldenströms Gata 35, SE-214 28 Malmö, Sweden

**Keywords:** PRDM15, PRDM15 substrates, PRDM15 gene effect, methyltransferase, methyl transfer

## Abstract

The PRDM family of methyltransferases has been implicated in cellular proliferation and differentiation and is deregulated in human diseases, most notably in cancer. PRDMs are related to the SET domain family of methyltransferases; however, from the 19 PRDMs only a few PRDMs with defined enzymatic activities are known. PRDM15 is an uncharacterized transcriptional regulator, with significant structural disorder and lack of defined small-molecule binding pockets. Many aspects of PRDM15 are yet unknown, including its structure, substrates, reaction mechanism, and its methylation profile. Here, we employ a series of computational approaches for an exploratory investigation of its potential substrates and reaction mechanism. Using the knowledge of PRDM9 and current knowledge of PRDM15 as basis, we tried to identify genuine substrates of PRDM15. We start from histone-based peptides and learn that the native substrates of PRDM15 may be non-histone proteins. In the future, a combination of sequence-based approaches and signature motif analysis may provide new leads. In summary, our results provide new information about the uncharacterized methyltransferase, PRDM15.

## 1. Introduction

Transcription factors (TFs) are sequence-specific DNA-binding regulators, which coordinate the transcriptional program downstream of multiple signaling pathways, ultimately shaping division, growth, and death of eukaryotic cells [1,2]. Dysregulated transcriptional programs are associated with a plethora of diseases, notably cancer [3,4]. TFs are desirable targets for cancer therapy for several reasons: (1) their dysregulation tends to occur in early stage tumorigenesis; (2) perturbation of TF-driven transcriptional programs can block differentiation of cancer cells and prevent cell death of cancer cells; and (3) TFs play a pivotal role in the establishment and maintenance of oncogenic gene expression networks; thus, it is more difficult for cancer cells to bypass their inhibition/inactivation by activating alternative pathways [5,6,7,8].

The PRDM (PRDI-BF1 and RIZ homology domain containing) family of TFs, as a subtype of the protein methyltransferase (PMT) family, is attractive from a therapeutic perspective because of the presence of C2H2 zinc fingers (ZFs) at the *C*-terminus, which confer sequence specificity, and a PR domain at the *N*-terminus, which confers potential enzymatic activity and tractability [9,10]. The PR domain is functionally and structurally related to the SET (suppressor of variegation 3–9, enhancer of zeste and trithorax) domain, which is the catalytic domain of protein lysine methyltransferases. Additionally, inhibitors of PMTs are being pursued intensely by both academic institutions and the pharmaceutical industry as precision cancer therapeutics [11,12,13,14]. Indeed, some, but not all, PR domain PMTs have been shown to directly methylate lysine residues. Similar to some of the understudied kinases, PRDMs belong to an extremely interesting, yet largely uncharacterized, class of methyltransferases [10].

Each of the 19 PRDM family members is a unique transcription factor, binding to a specific motif on chromatin, and there seems to be little overlap in function between the different family members [9]. Nonetheless, many PRDMs are involved in cancer initiation and/or maintenance, such as (i) PRDM1, a tumor suppressor in diffuse large B cell lymphoma (DLBCL) and other hematological tumors [15,16]; (ii) PRDM2, which is frequently deleted or rearranged in multiple cancer types; (iii) PRDM5, which is frequently silenced in multiple types of cancer [17]; (iv) PRDM14, a unique epigenetic regulator, upregulated in nearly 25% of human lymphoid neoplasms [18]; and (v) PRDM15, which we have observed to be overexpressed in human lymphomas [19,20,21].

We previously characterized PRDM15 as an important TF during development, but it is largely dispensable in adult mouse homeostasis [19]. Recently, we described the function of PRDM15 in sustaining lymphomagenesis [19]. Consistent with this, PRDM15 has been proposed to be an excellent target for lymphoma therapy and a novel strategy for therapeutic intervention to selectively kill PRDM15-overexpressing tumors. However, so far, the endogenous substrate(s) of PRDM15 have not been identified, which leaves a knowledge gap and hampers the understanding of the fundamental functions of PRDM15.

**Figure 1:**
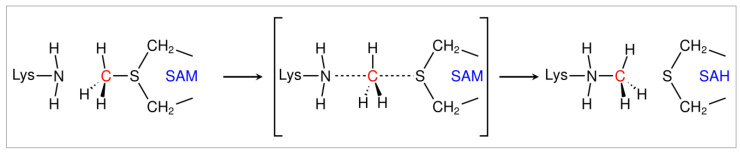
The reaction scheme of methyl transfer in PRDM9.

PRDM9, as one of the few PRDM methyltransferases with a well-determined X-ray structure and well-defined activities, is one of the best studied members of the PRDM family. PRDM9 displays high activity in catalyzing all the three states (mono-, di-, and tri-methylation) of H3K4 and H3K36. The SAM-dependent lysine methyltransferases employ two catalytic steps: (i) the deprotonation of the methyl lysine for the forthcoming methyl transfer and (ii) the methyl transfer. The deprotonation of the methyl lysine has been elusive but has been extensively discussed in our previous work [22]. Tyr357 was highlighted as a potential general base for the deprotonation of lysine, as its pKa is low enough to make it an ideal candidate for proton abstraction from the methyl lysine. Furthermore, Tyr357 was revealed to bridge two proton tunneling states which facilitate proton transport from Tyr357 to hydroxides, and the deprotonated Tyr357 is ready to take a proton from the methyl lysine [22]. For the methyl transfer, a conventional S_N_2 mechanism (see Figure 1) is well established. The breaking of the C–S bond and the formation of the C–N bond occur simultaneously through a transition state in which the carbon from the methyl group of SAM is pentacoordinated under an in-line nucleophilic attack, and is almost *sp*^2^ hybridized. The methyl group of SAM (CH_3_-SAH, with SAH referred to as the leaving group), is transferred to the N_ε_ of the deprotonated methyl lysine at 180°. The leaving group (SAH) is then withdrawn to the opposite side and the methylated lysine is formed with inversion of the tetrahedral geometry at the methyl group.

In summary, inferred from the reaction of PRDM9, we are hypothesizing that if PRDM15 is a methyltransferase: (1) the deprotonation happens before the methyl transfer (MT); (2) during the deprotonation stage, the catalytic competent distance between the ε-amino group of substrate lysine and the oxygen of Tyr should be around 3 Å; and (3) for the methyl transfer, the ideal distance between the sulfonium of SAM and the ε-amino group of the substrate lysine residue should be around 4.4 Å.

Non-Hodgkin’s lymphomas (NHLs) account for 90% of lymphomas [23]. This is a group of blood malignancies featuring a high diversity in terms of occurrence and prognosis [24]. The conventional therapy has a reasonable response rate (up to 50%); however, a significant proportion of NHL patients eventually relapse, and the expected survival is less than 1 year for some patients [24]. There is a need to identify new strategies for the treatment of NHLs. PRDM15 emerges as a potential target as it is dispensable in normal cells, but critical in sustaining B-cell lymphomagenesis. We hypothesize that targeting PRDM15 will have fewer side effects and will increase the specificity. The latest progress shows that PRDM15 regulates the transcription programs that involve the PI3K/AKT/mTOR pathway and glycolysis in B-cell lymphomas [19]. However, detailed information about PRDM15’s interactions and structure at the molecular level are missing, which limits the understanding of its function. Taken together, there is an unmet need to identify the functions of PRDM15 for therapeutic intervention across a wide range of aggressive lymphomas [19]. Is it possible to use computational methods to identify its endogenous substrates and further elucidate the reaction mechanism of this uncharacterized methyltransferase? Here, we will present our exploratory work to address this question.

## 2. Results

### 2.1. PRDM15 Dependency, Expression, and Associations

**Figure 2:**
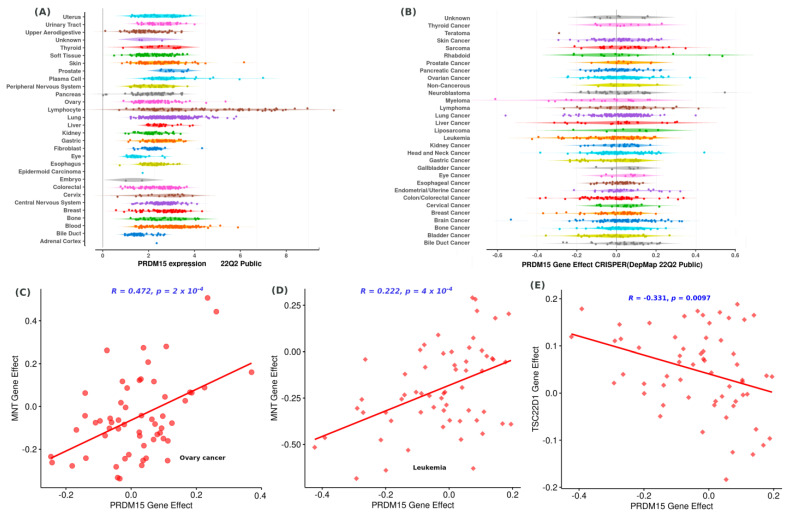
Examination of the cancer cell dependency on PRDM15 gene knockout from the DepMap data. (**A**) PRDM15 is overexpressed (*x*-axis indicates the expression log2 value) in all cell lines, particularly lymphocyte cell lines. (**B**) PRDM15 knockout decreases the proliferation of cancer cells. (**C**) Ovary cancer shows co-dependence on PRDM15 knockout and knockout of MNT. (**D**) Leukemia cancer displays co-dependence on PRDM15 knockout and MNT knockout, and (**E**) TSC22D1 gene.

Large-scale research initiatives have been launched to unravel cancer vulnerabilities. One such endeavor is the Dependency Map (DepMap), which uses genome-wide CRISPR and RNAi loss-of-function screens in hundreds of cancer cell lines to identify essential genes for proliferation. By exploring the ongoing DepMap project (22Q2), we have found that PRDM15 is overexpressed in almost all cancer cell lines, particularly in lymphoma cell lines (see Figure 2) and multiple myeloma cancer lines which display relatively higher dependence on PRDM15 compared to other cancer lines (Figure 2B). 

An exemplary analysis of the two PRDM15 co-dependency genes MNT and RALGDs revealed that it is correlated with MNT in both ovary cancer cell lines (Figure 2C) and blood cancer cell lines (Figure 2D) and negatively correlated with TSC22D1 in blood cancer cell lines (Figure 2E).

**Figure 3:**
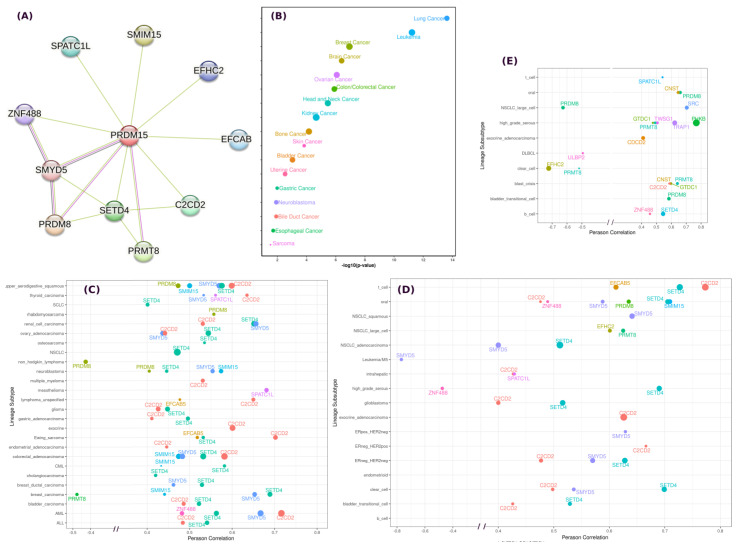
(**A**) The predicted protein-PRDM15 associations, both functional and physical, are indicated by edge. This figure was generated using the STRING web server. (**B**) SETD4 co-expression profile with PRDM15. The x-label indicates the *p*-value significance. The size of the balls indicates the Pearson correlation of the PRDM15 expression and SETD4 expression across different cancer cell lines. The expression data are based on Expression 22Q2 Public. (**C**) The expression of these genes that are associated with PRDM15 were further analyzed based on Lineage subtype and subsubtype (**D**). (**E**) The correlation between PRDM15 expression and the methylation profile of associated genes. Note that the *x*-axis only shows a Pearson Correlation of less than −0.4 and larger than 0.4, while *y*-axis indicates the lineage subsubtype. The size of the dots indicates the statistical significance based on the −log10(*p*-value) with a cutoff of *p* < 0.05 and the number of samples is more than 10.

In an attempt to infer the PRDM15 gene network, we have analyzed all cell lines. Using the top 100 co-dependency genes identified by CRISPR (DepMap 22Q2 Public+Score, Chronos), we first used pathway enrichment analysis (PEA) [25] to analyze these genes and found that they are involved in many pathways. Using prior knowledge, we have examined the pathways involving PI3K/AKT, gluconeogenesis, and regulation of beta-cell development. Among them, HPRT1, TSC22D1, and COX16 have significantly correlated dependence profiles (*p* < 0.05) with PRDM15 in lymphocyte or blood cancer cell lines from these pathways. Using the known and predicted protein–protein associations integrated and transferred across organisms [26], also known as STRING, we have plotted the genes that have functional and physical associations with PRDM15 (Figure 3A). Among these genes, SETD4 shows a strong co-expression profile with PRDM15. Figure 3B shows the *p*-value of the co-expression of SETD4 and PRDM15 across different cancer types, and a great significance is observed among many cancer cell lines. The expression of other genes that were predicted to be associated with PRDM15 were analyzed further based on the Lineage subtype and subsubtype. SETD4 and CDCD2 stand out as two genes which have the strongest co-expression profiles (Figure 3C,D).

Furthermore, based on the transcription_start_site [methylation (1 kb upstream TSS)), we analyzed the correlation between PRDM15 expression and the methylation of the associated genes predicted by both STRING and dependence probability. The dependency probability is based on CRISPR (DepMap 22Q2 Public + Score and Chronos) and RNAi screening data (Achilles+DRIVE+Marcotte and DEMETER2). We found that in high grade serous lineage, PHKB, TRAP1, TWSG1, PRMT8, and GTDC1 have stronger correlation in their DNA methylation profile with PRDM15 expression.

### 2.2. PRDM15 Native Substrates beyond Histone-Based Peptides

In order to identify PRDM15 substrates, our first-round modeling was based on H3K4 substrates. 1000 comparative models were generated and based on (i) 3IHX.pdb and 4C1Q.pdb templates (500 models generated) and (ii) the 4C1Q.pdb template (500 models generated). For the models generated based on the 3IHX and 4C1Q templates, supported on our previous QM calculation of methyl transfers [22], the ideal distance between the sulfonium and the ε-amino groups of the substrate lysine residues is around 4.4 Å, which serves as the first criteria in selecting 56 catalytic-possible models out of 500. Although the two signature motifs in the SET domain methyltransferase substrates consisting of ELxF/YDY and NHS/CxxPN (x is any amino acid) are conserved, they are not well conserved in the PRDM family. In PRDM9, AdoMet binds to Asn320, which is the only residue of the NHS/CXXPN motif (_320_NCARDDEEQN in mPRDM9) necessary for cofactor binding. However, the _320_NCARDDEEQN in mPRDM9 is replaced with the _147_RPALEPGH in mPRDM15, which provides another criterion to detect structures in which Arg147 has direct contact with the cofactor AdoMet.

**Figure 4:**
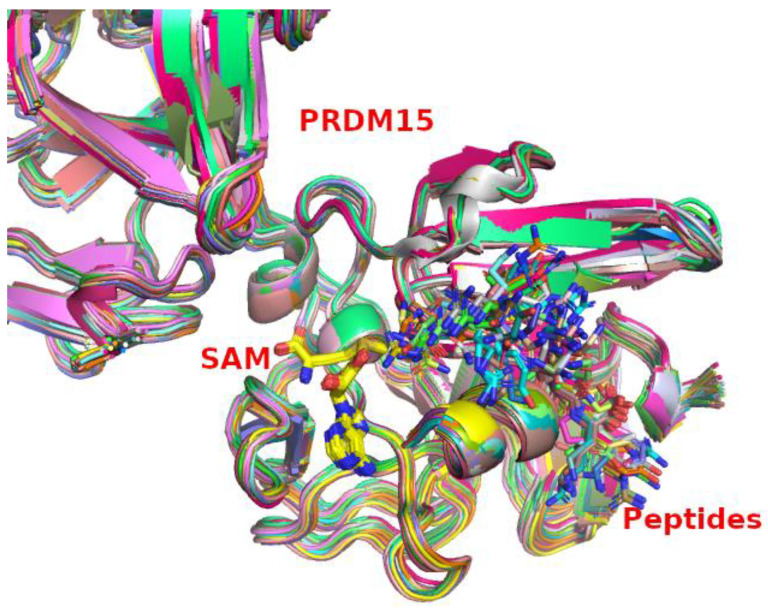
Superimposition of the modeled PRDM15 complex with 64 different peptides and SAM.

Our second-round modeling was based on potential histones substrates, which leads us to 64 peptides with a length of 7 amino acids (see Table 1) from H1.2, H2B, H3.1, and H4. The complex of PRDM15 with histone-based peptides and SAM is shown in Figure 4. We calculated the binding energy of these 64 peptides with the PDLD-S/LRA method and the data is shown in Table 1. The top-ranked six peptides are listed in Table 2. In addition, we have also removed the histone-based peptides methylated by PRDM9 and calculated the binding energy as a reference. The binding energy of PRDM9 and its natural substrate is −2.73 k_cal_/mol and its natural substrates were ranked in the top 20 of the 64 peptides repertoires. Our preliminary investigation of the histone-based peptides indicated a need to expand our investigation to non-histone-based peptides, and there is an implication that the native substrates of PRDM15 could be non-histone proteins.

### 2.3. PRDM15 Potential Methylation Profile

In our previous work [22], we found that the pKa of Tyr357 in PRDM9 is quite low and the low pKa of tyrosine makes it an ideal candidate to withdraw protons from the methyl lysine through shared low-barrier H-bond. Since there are three tyrosine residues in the active site of PRDM9 and they are partially deprotonated, they are capable of changing their H-bond pattern, thus bridging two low-barrier proton tunneling states and providing a cascading proton transfer from methyl lysine to the hydroxides. In the end, we proposed that the three tyrosines generate three states of deprotonated lysine which renders the mono-, di-, and tri-methylation of lysine feasible.

More than 200 protein methyltransferases have been discovered so far [27], among them more than 50 are human lysine histone methyltransferases (KHMTs) [28]. Approximately 50 protein lysine methyltransferases (PKMTs) catalyze reactions that result in mono-, di-, and/or tri-methylated lysine residues on histone and nonhistone substrates [29].

Furthermore, we have analyzed the other three well-known enzymes from the SET domain families: G9a (also known as EHMT2), G9a-like protein (GLP; EHMT1), and SUV39H2. G9a and GLP catalyze the mono- and di-methylated states of histone H3K9 (H3K9me1 and H3K9me2) [30], and SUV39H2 causes the di- and tri-methylation of a mono-methylated lysine substrate [30].

**Figure 5:**
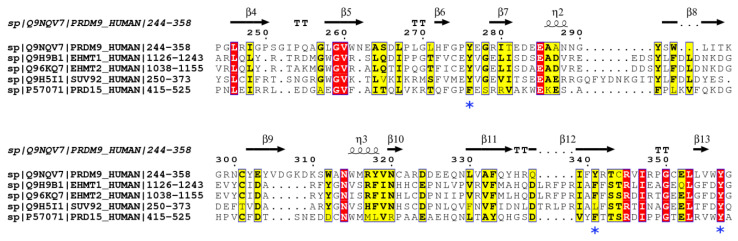
Sequence alignment of PRDM9, EHMT1 (GLP), EHMT2 (G9a), SUV92H2, and PRDM15. This figure was generated by ESPript 3.0. Note that the blue stars indicate the catalytically important residues.

The sequence alignment of PRDM9, GLP, G9a, SUV39H2, and PRDM15 is shown in Figure 5. GLP, G9a, and SUV39H2 have two tyrosines, corresponding to their two methylation activities, while PRDM9 has all three tyrosines conserved, which correspond to its three methylation activities. Since the current available information about the substrates of methyltransferases is scarce, the analysis of four examples may have limited statistical importance; however, together with mutation studies [31,32], the analysis suggests that mutation of these key tyrosine residues decreases catalytic activity. Furthermore, tyrosine has long been regarded as a key player in target methyl lysine proton abstraction [22,33], and the Tyr/Phe switch changes the product specificity [34]. Based on the above knowledge, we scrutinized the sequence alignment between PRDM15 and PRDM9. Since only one (Y357) of the three tyrosine residues (Y276, Y341, and Y357) at the active site is conserved in PRDM15 (see Figure 5), we predict that this one tyrosine residue is capable of only one deprotonation activity, rather than the three deprotonation activities as catalyzed by PRDM9. Hence, we hypothesize that the putative substrate(s) of PRDM15 can either have a mono-methylated lysine ready to be di-methylated, a di-methylated lysine to be tri-methylated, or that the lysine of the substrate can only be mono-methylated.

## 3. Discussion

### 3.1. PRDM15 as a Potential Drug Target

Transcription factors (TFs) bind to chromatin in a sequence specific manner to regulate unique transcriptional programs, which ultimately determine cell fate [2]. However, during tumorigenesis, deregulated signaling pathways result in aberrant gene expression programs, which underlie the block in terminal differentiation and the acquisition of increased proliferate capacity, a hallmark of cancer [1]. Inhibiting TFs would be an excellent therapeutic strategy. However, this has been challenging due to the lack of specific enzymatic activity, a huge interaction surface between TF and DNA, and the significant structural changes that occur upon DNA binding. The PRDM family of transcription regulators, as a subtype of the protein methyltransferase (PMT) family, encompasses an attractive class of TFs from a therapeutic perspective, given that their protein structure contains both DNA-binding zinc fingers (ZFs), conferring sequence-specific chromatin binding, and a PR domain, conferring enzymatic activity and tractability [4]. Additionally, inhibitors of PMTs are being pursued intensely by both academic institutions and the pharmaceutical industry as precision cancer therapeutics [5,6,7,8]. PRDM15 has recently been shown to be highly expressed in multiple cancer types and more specifically in B cell malignancies [4]. Given the diversity of B cell tumors, there is an unmet need to identify novel proteins that can be targeted for therapeutic intervention across a wide range of aggressive subtypes of B cell malignancies and other types of cancer.

In addition, PRDM15 is overexpressed in immune cells and follicular lymphoma [35] and is critical for maintenance of human lymphomas and embryonic development [19]. However, it is dispensable for normal adult murine homeostasis. PRDM15 depletion increases specificity in killing B cell lymphomas. Targeting PRDM15 leads to a metabolic crisis and eventually cell death [19]. PRDM15 has been suggested as a good target for B cell lymphoma treatment [19].

### 3.2. Outlook

Future directions include (1) to degrade and inhibit PRDM15; a virtual screening can be used to discover molecules which inhibit PRDM15’s action or we will develop a PROTAC for its degradation and (2) investigating its catalytic profile and the key residues that contribute to the catalytic cycle and their interactions with potential inhibitors. In order to block the functions of PRDM15 in a pathological context and develop a precision therapy for lymphoma and leukemia, mutations, variants, and other genetic polymorphisms of PRDM15 will be investigated.

## 4. Materials and Methods

### 4.1. Homology Modeling of PRDM15

**Figure 6:**
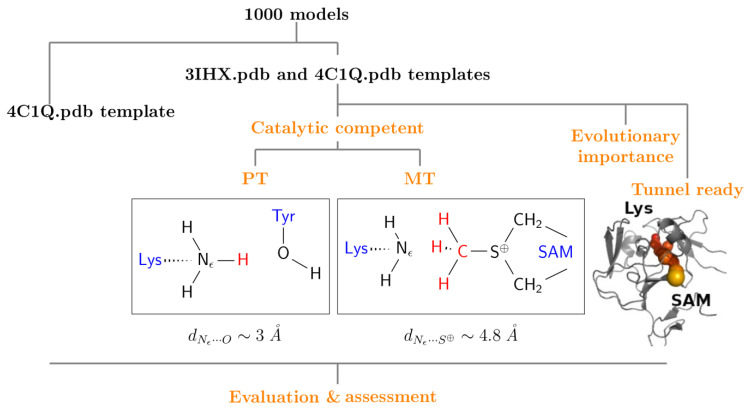
The selection procedure during the homology modeling of PRDM15.

There is no crystal structure available for PRDM15. Upon scrutiny of the currently available crystal structures of the methyltransferase domain of the enzymes from the same family as PRDM15 in the Protein Data Bank, such as 3DB5.pdb (human PR domain-containing protein 4) and 3DAL.pdb (human PR domain-containing protein 1), we notice that the structures without substrate/inhibitor binding (apo-structures) have elongated loops. Hence, the attempt to crystallize the PR domain of PRDM15 without the substrate might not be enough to completely understand its function. Our prediction is that substrate binding would induce significant conformational changes; hence, to successfully crystallize the PR domain of PRDM15, it is paramount to identify such substrate(s). Our theoretical approaches may contribute to advancing the knowledge of PRDM15 structures. We will start with the homology modeling of the PRDM15 structure with the pocket ready for substrate binding. Initially, the PR domain of the canonical protein sequence of PRDM15 (uniport ID: E9Q8T2) was used for the template search on the NCBI PSI-BLAST server to model the three-dimensional structure of PRDM15. The primary search led to PRDM10 (PDB ID: 3IHX) and PRDM9 (PDB ID: 4CIQ). Significant alignment is provided by 3IHX with an E-value of 4 × 10^−28^. Substrate (H3K4me2) and cofactor (SAH) binding pockets are provided by 4CIQ. PRDM15, PRDM10, and PRDM9 sequence alignments were performed on the Clustal Omega webserver at the European Bioinformatics Institute (GB). Our first modeling used the ARTKQTA peptide (N-terminus of histone H3) as the putative substrate; in addition, H3K4me2 was converted back to H3K4 by removing the two methyl groups, and SAH was converted back to AdoMet by adding one methyl group. A total of 1000 comparative models were generated by “automodel” within MODELLER 10.1. DOPE score3 was used for the initial evaluation. For the above models generated with 3IHX and 4C1Q templates, based on our previous study [22] of proton transfer (PT), the catalytic competent distance between the ε-amino group of substrate lysine and the oxygen of Tyr was around 3 Å. For the methyl transfer (MT), the ideal distance between the sulfonium of SAM and the ε-amino group of substrate lysine residue is around 4.4 Å. These two criteria served were used in our model selection. The evolution trace method was used to identify the evolutionary important residues in order to highlight functional hot spots [36]. The procedure of our modeling and selection is shown in Figure 6.

### 4.2. Theoretical Approach for the Search of Endogenous Substrate(s) of PRDM15 

Since the substrate(s) of PRDM15 are not known, one strategy is to identify substrate(s) starting from histone-based peptides and later explore non-histone targets, especially those involved in pathways regulated by PRDM15 [19]. A preliminary analysis of the peptides involved in the MAPK and Wnt pathway revealed 1152 potential methylation targets. We estimated that the peptide repertoire consists of approximately several thousand peptides. If we are to model PRDM15 with all these peptides in different methylation states (methyl lysine: Kme0; mono-methylated lysine: Kme1; and di-methylated: Kme2), this would generate tens of thousands of complexes. Peptides with a high binding affinity towards PRDM15 were selected based on the binding energy calculation either by the semi-macroscopic version of the protein dipole Langevin dipole with the linear response approximation (PDLD-S/LRA) [22], which has been well established to provide a reliable estimation of the binding free energies, or other methods. Our exploratory work started with histone-based peptides, with the search pattern “xxxKxxx” (x is any residue), there are 64 peptides containing Lys residues (see Table 1). For each peptide, we modeled 500 structures, and among them, the ‘best’ ones were selected based on the lowest MODELLER objective functions they have.

### 4.3. Binding Energy Calculations

The binding free energies of the potential substrates bound to PRDM15 were evaluated by the semi-macroscopic version of the protein dipole Langevin dipole (PDLD) with the linear response approximation (PDLD-S/LRA). At first, we generated PRDM15 peptide SAM complex configurations with the charged and uncharged forms of solute, and then treated the long-range interaction with the local reaction field (LRF) [37]. After our explicit all-atom molecular dynamics simulations with the surface-constrained all-atom solvent (SCAAS) [38], we carried out PDLD/S calculations on the generated configurations. We took the average value from these generated configurations as a consistent estimation of the binding free energy. In total, we generated four configurations for the charged and uncharged states, respectively. A 2 ps run was performed for each of these simulations at 300 K. First, we evaluated the binding energy of the complexes selected for our 64 histone-based peptides. Based on previous work [22,39], we estimated the intrinsic binding energy with a dielectric constant of four for neutral protein, and an effective dielectric constant of 60 for the charge–charge interaction between the substrate, cofactor, and the side chains of the ionizable residues.

### 4.4. Statistics Analysis

The expression data and dependency scores were obtained from DepMap Project datasets (DepMap 22Q2 Public + Score, Chronos). First, we imported these DepMap data into the Rstudio2022.07.1+554 environment, and then they were analyzed using tools within R. The visualization of the data was accomplished within R using ggplot2 and dplyr libraries.

## Figures and Tables

**Table 1:** The binding energy between 64 histone-based peptides and PRDM15.

ID	Peptides	Binding Energy	ID	Peptides	Binding Energy
1	AALKKAL	1.52	33	KAAKKAG	1.04
2	AGAKKAV	1.83	34	KAAKPKA	1.41
3	AGVKKVA	−0.44	35	KAAKPKV	0.27
4	AKAKKPA	0.41	36	KATKKAA	2.64
5	AKPKAAK	0.7	37	KDGKKRK	0.06
6	AKPKKAT	0.48	38	KPKKAAK	−0.46
7	ALKKALA	0.44	39	KSPKKAK	1.75
8	APKKGSK	0.03	40	KTPKKAK	2.1
9	ARAKAKT	0.74	41	LATKAAR	0.39
10	ARTKQTA	−1.42	42	LGLKSLV	1.73
11	ATPKKAK	2.09	43	LIRKLPF	2.63
12	ATPKKSA	−1.3	44	LITKAVA	0.19
13	AVTKAQK	0.33	45	LLRKGNY	0.44
14	DVEKNNS	1.62	46	PAEKAPV	−0.75
15	EGTKAVT	0.74	47	PKAKKAG	0.14
16	EHAKRKT	2.55	48	PVEKSPA	0.72
17	ELAKHAV	1.04	49	QDFKTDL	1.9
18	ELNKLLG	0.56	50	RDNKKTR	1.85
19	EPAKSAP	−0.07	51	RHRKVLR	2.88
20	GAAKKPK	1.86	52	RSRKESY	1.95
21	GAAKRKA	1.15	53	RYQKSTE	1.48
22	GEAKPKV	2.49	54	SAAKAVK	0.85
23	GGTKPKK	2.4	55	SHHKAKG	2.77
24	GGVKKPH	−1.66	56	SPAKPKA	1.04
25	GGVKRIS	1.46	57	TGGKAPR	0.71
26	GITKPAI	1.36	58	TPRKASG	2.31
27	GLGKGGA	1.09	59	TVTKKVA	−0.3
28	GRGKGGK	0.32	60	VKPKAAK	−0.35
29	GRGKQGG	1.3	61	VKPKKAA	1.08
30	GSFKLNK	0.22	62	VQTKGTG	1.84
31	HYNKRST	2.75	63	YALKRQG	2.84
32	IHAKRVT	2.36	64	YVYKVLK	3.44

**Table 2:** The top-ranked six peptides from our 64 peptide-repertoire.

PRDM15	Histone	Peptides	Binding Energy
	H3K37	GGVKKPH	−1.66
	H3K5	ARTKQTA	−1.42
	H1.2K148	ATPKKSA	−1.3
	H1.2K17	PAEKAPV	−0.75
	H1.2K184	KPKKAAK	−0.46
	H1.5K168	AGVKKVA	−0.44
PRDM9	H3K4	ARTKQTA	−2.73

## Data Availability

The DepMap data was accessed from https://depmap.org/portal/ on 12 December 2022.
